# Understanding mechanistic relationships between IgG titers and Fc effector functions: a computational framework to assess polyfunctionality

**DOI:** 10.3389/fimmu.2025.1578500

**Published:** 2025-09-16

**Authors:** Suzanne K. Shoffner-Beck, Robert M. Theisen, Kade E. Wong, Supachai Rerks-Ngarm, Punnee Pitisuttithum, Sorachai Nitayaphan, Stephen Kent, Amy W. Chung, Kelly B. Arnold

**Affiliations:** ^1^ Department of Biomedical Engineering, University of Michigan, Ann Arbor, MI, United States; ^2^ Department of Disease Control, Ministry of Public Health, Bangkok, Thailand; ^3^ Vaccine Trial Centre, Faculty of Tropical Medicine, Mahidol University, Bangkok, Thailand; ^4^ Armed Forces Research Institute of Medical Sciences, Bangkok, Thailand; ^5^ Department of Microbiology and Immunology, The Peter Doherty Institute for Infection and Immunity, The University of Melbourne, Melbourne, VIC, Australia; ^6^ Melbourne Sexual Health Centre and Department of Infectious Diseases, Alfred Health, Central Clinical School, Monash University, Melbourne, VIC, Australia

**Keywords:** antibody-mediated effector functions, antibody-dependent cellular cytotoxicity (ADCC), human immunodeficiency virus (HIV), ordinary differential equation model, mechanistic model

## Abstract

**Introduction:**

Recent vaccine and infectious disease studies have highlighted the importance of antibodies that activate cellular Fc functions, including antibody-dependent cellular phagocytosis (ADCP) and antibody-dependent cellular cytotoxicity (ADCC), which are mediated by different Fc gamma Receptors (FcγRs). Activation of these functions requires complex overlapping interactions between IgG antibodies, FcγRs, and antigens that can be challenging to deconvolve experimentally.

**Methods:**

Here we created an ordinary differential equation model that simultaneously predicted FcγRIIIa immune complexes upstream of ADCC and FcγRIIa immune complexes upstream of ADCP as a function of antigen, IgG, and FcγR concentration and binding properties. We then used the model to dissect mechanisms driving immune complex formation.

**Results:**

Model results suggested that the maximum formation of immune complexes would not occur at highest total IgG titers. Instead, higher IgG titers have the potential to decrease FcγRIIIa (ADCC) and/or FcγRIIa (ADCP) immune complexes, due to competition between antibody subclasses for antigen and FcγR binding. We used the model to simulate vaccine boosts of IgG1 or IgG3 in 105 participants from an HIV vaccine trial, and found that boosting IgG1 and IgG3 in combination was not predicted to result in significant changes in either FcγRIIIa (ADCC) or FcγRIIa (ADCP) immune complexes. Surprisingly, simulated boosting of IgG3 alone had the potential to significantly decrease ADCP (p<0.00001), though it would increase ADCC responses. We also illustrated how the model could be used to assess how variability in viral load, FcγR expression, FcγR polymorphisms, and IgG titers across different tissue compartments can lead to differences in FcγRIIIa and FcγRIIa complexes.

**Discussion:**

Altogether, these results illustrate how a computational framework provides new quantitative insights into activation of Fc effector functions that could be used to guide future rational design of therapeutic and prophylactic interventions.

## 1 Introduction

Recent vaccine and infectious disease studies emphasize the importance of non-neutralizing antibody functions that can activate Fc Receptors (FcRs) on innate immune cells to induce multiple cellular functions including antibody-dependent cellular phagocytosis (ADCP) and antibody- dependent cellular cytotoxicity (ADCC) (1–6). These functions have been correlated with protection and linked to optimal vaccine efficacy for a broad range of pathogens. For example in HIV, ADCC was identified as a secondary correlate of protection in the RV144 vaccine trial, and linked to increases in vaccine-specific IgG1 and IgG3 (6–9). In the same RV144 study, polyfunctional antibodies capable of activating multiple Fc-mediated responses were induced, similar to those seen with protective non-human primate HIV vaccines and in HIV elite controllers (10–13). In contrast, monofunctional antibodies were observed in non-protective HIV vaccines (6), though it has not been possible to replicate these results in vaccine trials conducted in other populations. Altogether these findings underscore the importance of understanding quantitative mechanisms by which ADCC, ADCP, and other Fc effector functions are activated, especially person-to-person variability driven by differences in antibody titers and binding properties. Such insights would be valuable for tailoring effective immune interventions against pathogens in diverse populations.

ADCC and ADCP share overlapping upstream activators, relying on the binding of IgG antibodies to FcγRs (traditionally FcγRIIa for ADCP and FcγRIIIa for ADCC). However, the activation of these pathways is complex and influenced by several factors, including individual variation in IgG subclass titers (IgG1–4), post translational modifications (such as glycosylation of the antibody Fc region), such as glycosylation of the antibody Fc region), and genetic polymorphisms in FcγRs. Additionally, tissue-specific differences in immune cell populations and receptor expression, such as FcγRIIa on phagocytes and FcγRIIIa on natural killer (NK) cells, drive heterogeneity in Fc effector responses. This heterogeneity is particularly pronounced in mucosal environments like the respiratory and gastrointestinal tracts, where distinct immune populations and local antibody concentrations shape immune responses. Challenges associated with evaluating human tissue-specific samples limit the ability to experimentally assess the relative importance of individual changes in this complex system.

Vaccine boosting strategies have been employed to enhance neutralizing antibody titers, but their effects on Fc effector functions remain less understood. In HIV, boosting has been shown to elevate total IgG levels but skew subclass distribution toward IgG2 and IgG4, leading to diminished Fc effector activity (6, 14–16). Previous computational efforts have provided insight into FcR activation and ADCC/ADCP dynamics. For instance, Lemke et al. (2021) developed an ordinary differential equation (ODE) model to examine personalized responses in RV144 participants (17). However, this model was limited in that it only evaluated complexes for each FcγR (FcγRIIa or FcγRIIIa) in isolation and did not consider the competition between FcRs for the same antibody-antigen complexes, which would be essential for understanding polyfunctionality.

In this study, we address these gaps by developing a new computational framework that models the simultaneous activation of FcγRIIa and FcγRIIIa. Our approach enables quantitative evaluation of the balance between ADCC and ADCP under varying conditions, such as changes in IgG subclass titers following vaccine boosting. Incorporation of interactions between multiple FcRs enables understanding of how genetic variability, antibody features, and tissue-specific environments contribute to polyfunctional immune responses. Furthermore, we illustrate how the model can be used to simulate differences that may arise in mucosal tissue compartments, providing insights that are difficult to obtain experimentally. Overall, this framework aids in unraveling the complexities of Fc-mediated polyfunctionality and the future design of immunotherapeutic strategies tailored to individual and tissue-specific immune landscapes.

## 2 Results

### 2.1 A sensitivity analysis highlights the importance of IgG1-and IgG3-related parameters, and how high titers may reduce Fc effector functions

**Figure 1:**
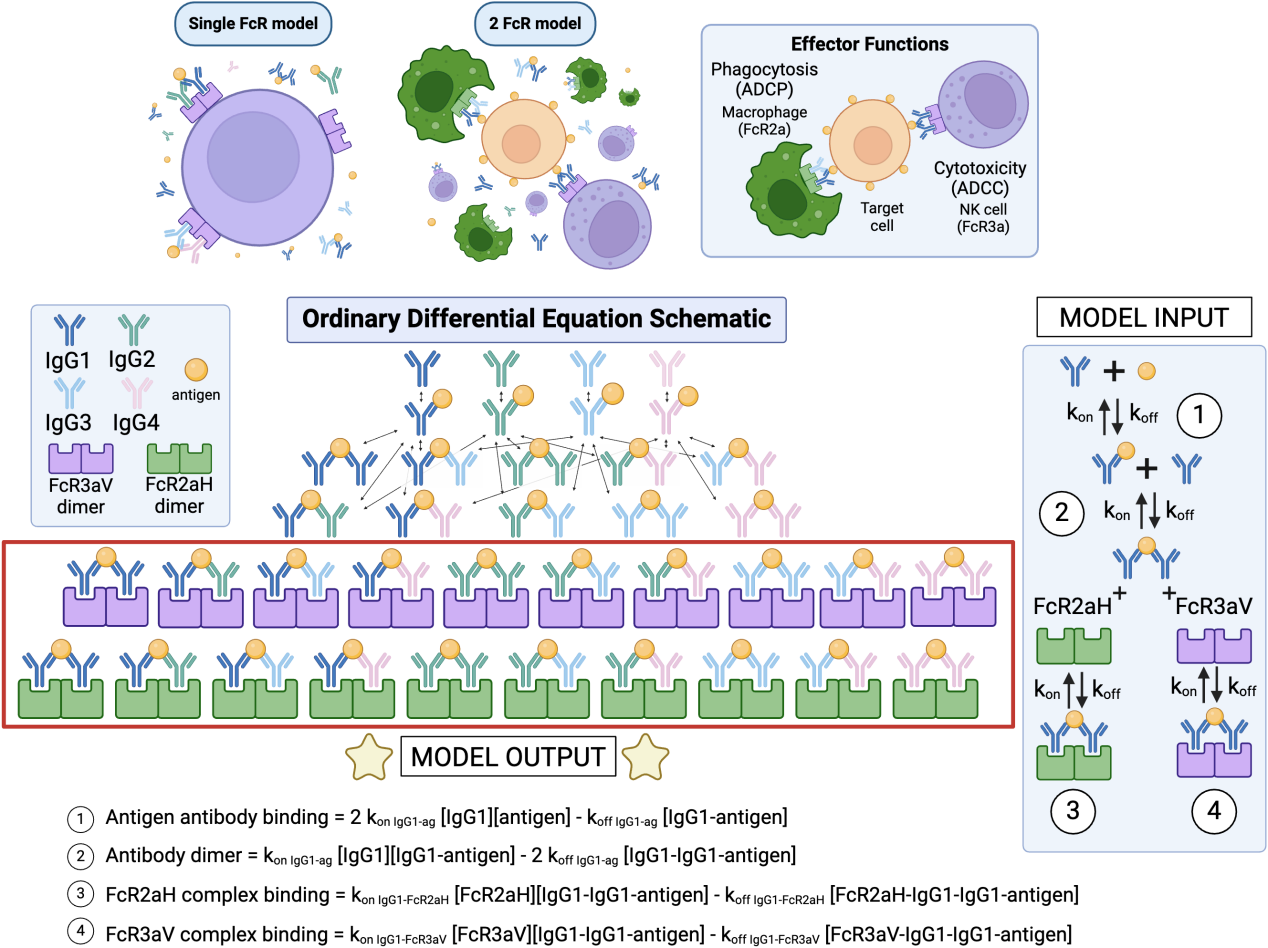
Model schematic illustration of FcR2a on macrophages and FcR3a on natural killer cells as examples for antibody-dependent cellular phagocytosis (ADCP) and antibody-dependent cytotoxicity (ADCC) respectively. Forward and reverse reactions shown for binding of antibody to antigen, formation of a dimer, then subsequent binding of either FcR2a or FcR3a.

We first created a system of ordinary differential equations (ODEs) to simulate the dynamic interactions between IgG antibodies, antigens, and multiple Fcγ receptors (FcγRs) in the blood (**Figure 1**). Example equations are provided in **Figure 1**, and a full list of equations can be found in **Supplementary Material**. The model extends our prior work in Lemke et al. (2021) (17) and Lemke et al. (2022) (18), by including multiple FcRs in parallel, which enables assessment of potentially competitive interactions that occur between multiple Fcγ receptors. This competition is central to polyfunctionality, allowing for better evaluation of differences in tissue-specific activation. The affinity parameters for the model are summarized in **Supplementary Table 1**. The forward binding rates of FcγR to the various IgG subclasses are taken from Bruhns et al. (19), the reverse binding rate is estimated from the average value in pooled RV144 samples, and the antigen-antibody binding rate is determined from SPR measurements from pooled samples. The concentrations of antibody, antigen, and FcγR for the blood were estimated from the literature (20–23) and are summarized in **Supplementary Table 2**.

**Figure 2:**
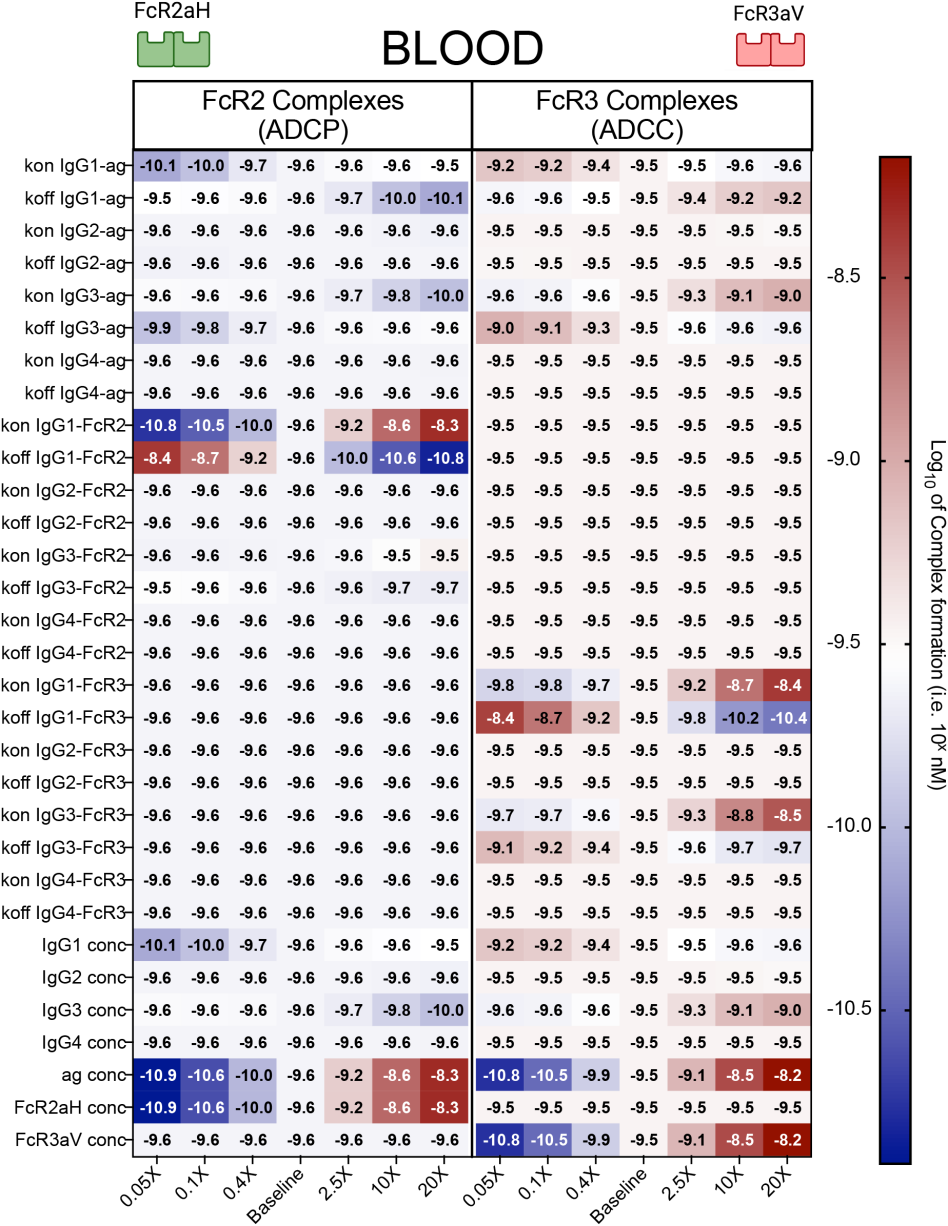
1D sensitivity analysis of two-FcγR model using *in vivo* blood parameters. Parameters were altered 0.05-20X of the baseline blood values (from **Supplementary Tables 1**, **2**) as the model was used to predict formation of FcγRIIa (left) and FcγRIIIa (right) complexes. Color indicates the amount of each respective complex formed.

In order to understand which IgG and FcR features have the most significant impact on FcγRIIa and FcRγIIIa complex formation, we performed a one-dimensional sensitivity analysis, in which each baseline parameter was varied individually by a factor of 0.05-20x and FcγRIIa and FcRγIIIa complex formation were subsequently calculated (**Figure 2**). Unsurprisingly, the most sensitive parameters for both FcγR outputs involved IgG1 and IgG3, specifically IgG1 and IgG3 concentrations, and IgG1 and IgG3 affinities for both antigen and Fc receptor. Antigen and Fc receptor concentration were also found to be sensitive parameters. In terms of IgG subclass concentrations, the model illustrated that increases in FcγRIIa complex formation were primarily associated with increases in IgG1, whereas increases in FcγRIIIa complex formation were driven by increases in IgG3. Both of these results were expected given the higher affinity of FcγRIIa for IgG1 and FcγRIIIa for IgG3. Intriguingly, however, the model also suggested that IgG1 and IgG3 have the potential to negatively impact FcγRIIIa and FcγRIIa complex formation respectively. When IgG1 was increased by 20-fold, the model predicted a 26.6% decrease in FcγRIIIa complexes. Likewise, the model suggested that a 20-fold increase in IgG3 may decrease FcγRIIa complex formation by as much as 54.9%. We also performed global sensitivity analyses, in which all parameters are varied simultaneously across a given range and parameter sets are chosen randomly within the parameter space across multiple simulations. The global sensitivity analysis revealed similar results, highlighting the importance of IgG1 and IgG3 concentration and affinity parameters and suggesting that increasing IgG1 decreases FcγRIIIa responses and IgG3 decreases FcγRIIa responses (**Supplementary Figure 1**). Inspection of the model revealed that the unexpected decreases in FcR complex formation with increased in IgG titers was likely due to competition between IgG1 and IgG3 subclasses for antigen, with stronger versus weaker binding for FcγRIIIa vs. FcγRIIa. At very high IgG1 concentrations, antigen remains bound in anti-IgG1 intermediate complexes, thus reducing antigen availability for IgG3 and lowering formation of FcγRIIIa complexes. A similar mechanism was observed for IgG3 and FcγRIIa. Overall, these results provide a mechanism by which increased levels of IgG1 and IgG3 may negatively impact FcγR complex formation and downstream activation of ADCC and ADCP. Interestingly, the sensitivity analysis also indicated that complex formation for each receptor was not sensitive to the concentration of the other receptor (**Figure 2**). This suggests that competition between FcγRs does not influence output; rather, it is driven by IgG subclass competition as described above. To explore this in more detail, we computed complex formation for each individual receptor alone (like the model framework created in Lemke et al., 2021 (17)), and also in the presence of the other receptor (**Supplementary Figure 5**). Results confirmed the absence of competition between receptors, as output for an individual receptor was the same, whether or not another receptor was included in the model. In other parameters spaces well outside the physiologically relevant range for this study (very high antigen concentration and very high FcR concentrations), we did find that FcγR competition has the ability to influence output (**Supplementary Figure 6**).

**Figure 3:**
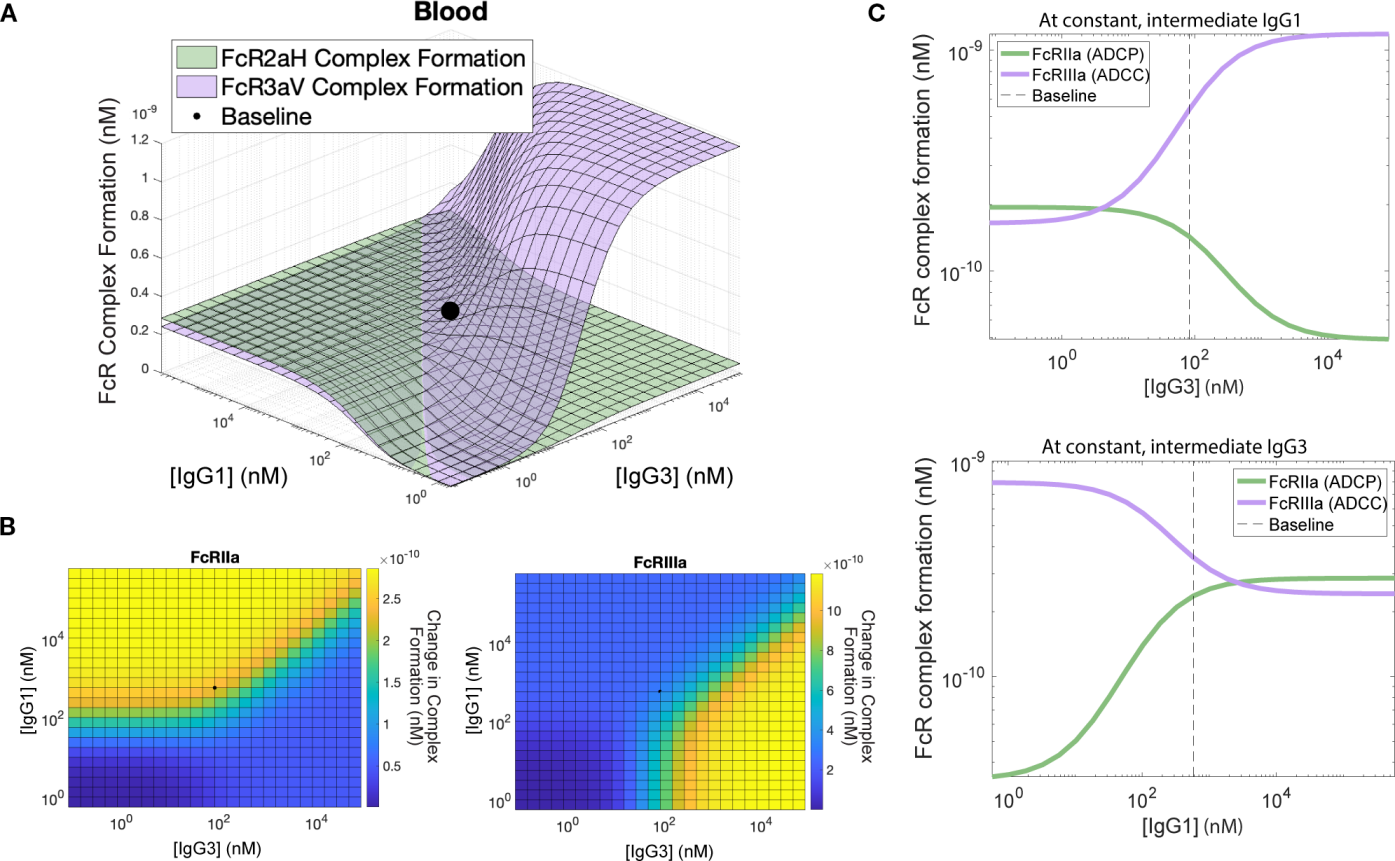
2D Sensitivity analysis and relationship between IgG1 and IgG3. **(A)** FcγRIIaH complex formation (green surface) and FcγRIIIaV (purple surface) plotted across a combination of IgG1 and IgG3 concentrations with baseline blood parameter values indicated with a black point. **(B)** Heatmap showing complex formation with yellow indicating higher values and blue indicating lower values. The curved line indicates the region with the highest gradient (regions which are very sensitive to small perturbations). **(C)** Cross-section of surface shown at constant IgG1 = 100nM (above) and constant IgG3 = 100 nM (below), showing the baseline x parameter value with a dashed line and the inverse relationship between FcγRIIaH formation and FcγRIIIaV formation as antibody levels increase.

In order to further explore the interaction between IgG1 and IgG3 and their combined effects on FcγRIIa and FcγRIIIa complex formation, we performed a two-dimensional (2D) sensitivity analysis by tuning each antibody concentration over .001x–1000x it's baseline value and computing complex formation for FcγRIIa and FcγRIIIa (**Figure 3A**). The resulting landscape highlighted changes in ADCP (FcγRIIa complexes) and ADCC (FcγRIIIa complexes) that might be expected with increases in IgG1 and IgG3 that result from vaccine boosting. The model suggested that more antibody is not necessarily ideal and that maximizing one Fc effector function tends to lead to decreases in another function at certain combinations of IgG1 and IgG3. For example, for FcγRIIa complexes, the landscape illustrates sensitivity to IgG1 up to 100 nM at low levels of IgG3 (<10^3^ nM), however this sensitivity has a limit and IgG3 has the capacity to reduce complex formation at high titers (**Figure 3A**). Conversely, FcγRIIIa complex formation is predicted to be highly sensitive to IgG3 from 10 to 500 nM, but at high levels of IgG1, FcγRIIIa engagement would be reduced (**Figure 3C**). Interestingly, at constant levels of IgG1, the model predicts that increases in IgG3 would increase FcγRIIIa complex formation (ADCC) but decrease FcγRIIa functions (ADCP). For example, a 1000-fold increase in IgG3 would increase ADCC by 616%, but in parallel it would be expected to decrease ADCP by 74.7%. This is likely due to the increased relative affinity of FcγRIIIa for IgG3 in comparison to FcγRIIa, which leads to competition between antibodies for antigen and less antigen-bound IgG1to activate ADCP via FcγRIIa. A similar effect was observed for constant IgG3, where increases in IgG1 were predicted to result in increases in FcγRIIa functions (ADCP) but decreases in FcγRIIIa functions (ADCC). For example, a 1000-fold increase in IgG1 leads to a 734% increase in ADCP while simultaneously leading to a 69.5% decrease in ADCC (**Figure 3**). Perhaps most interestingly, parallel increases in both IgG1 and IgG3 were not predicted to maximize FcγRIIa and FcγRIIIa complexes due to the potential for high IgG1 to significantly reduce FcγRIIIa complex formation.

**Figure 4:**
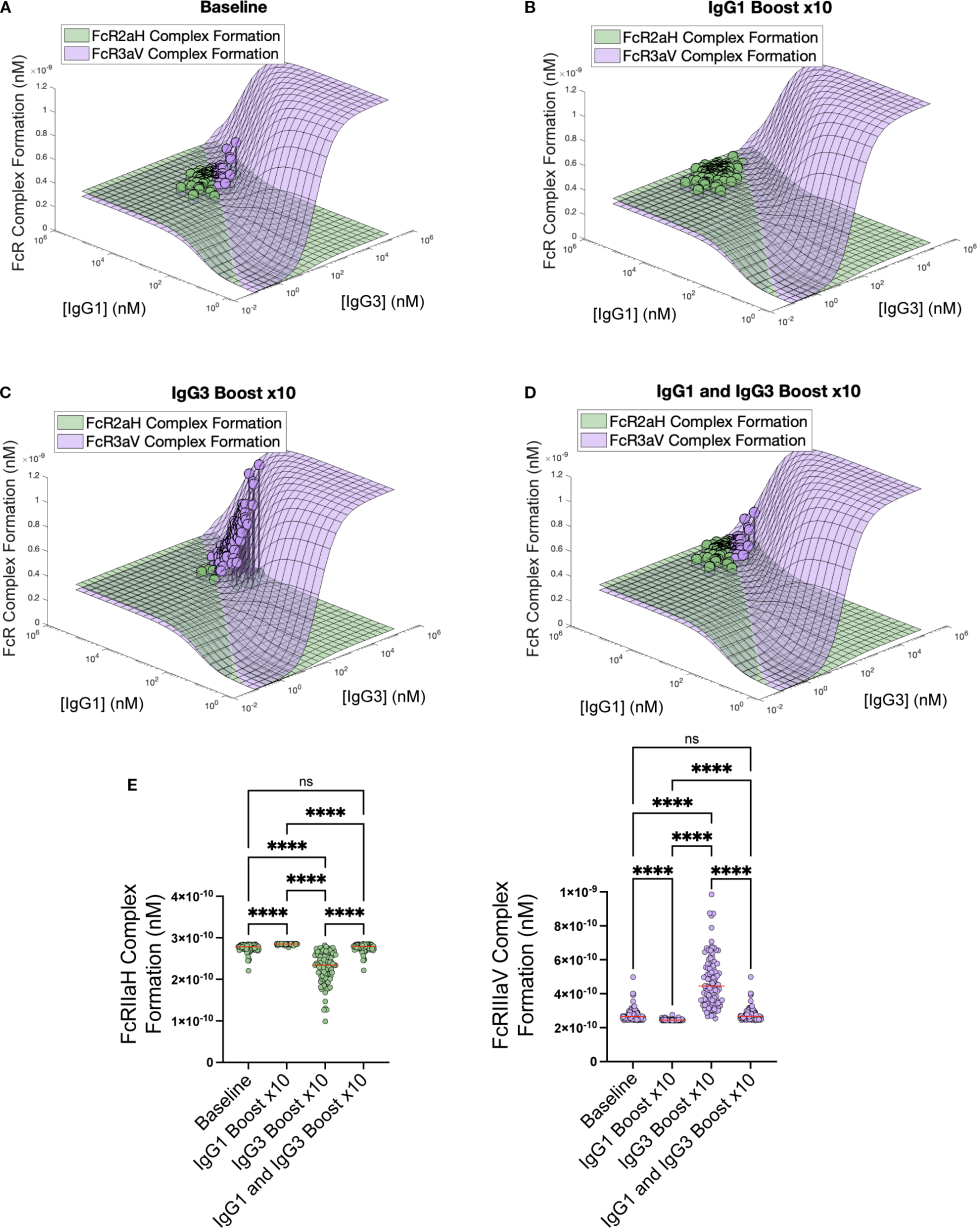
Simulation of the impact of antibody boosting in RV144 vaccinee data on FcR complex formation. **(A)** Baseline plasma values of gp120-specific IgG1 and IgG3 values with simulated FcR complex formation plotted on the surface. Simulated boosting of **(B)** IgG1-only x10, **(C)** IgG3-only 10x, and **(D)** simultaneous IgG1 and IgG3 boosting x10 plotted. **(E)** Statistical significance between the population FcγRIIa and FcγRIIIa formation across baseline and various interventions with significant adjusted p-value denoted by asterisks. Kruskal-Wallis with Dunn’s multiple comparison test was used to evaluate differences between antibody boosting regimens. (n.s.: p> .05, ****p < .0001).

To illustrate this concept more concretely in a vaccine population after boosting-related increase in IgG1 and IgG3, we used data from the RV144 HIV clinical trial, where antigen-specific IgG1, IgG2, IgG3, and IgG4, as well as antigen-specific FcγRIIa and FcγRIIIa responses were measured in 105 plasma samples. Experimental IgG subclass measurements made in mean fluorescent intensity (MFI units) were converted to concentration as described in the methods and previously published (Lemke 2021) (17). We used converted concentration values as input into our two FcR model and predicted *in vivo* blood FcγRIIa and FcγRIIIa complex formation for each vaccinee. The vaccinee samples are illustrated under *in vivo* conditions on the surface in **Figure 4A**, with an average FcγRIIa complex formation of 2.76e-10 nM and mean FcγRIIIa complex formation of 2.77e-10 nM. The model was then used to predict FcγRIIa and FcγRIIIa complex formation after a simulated “boost” in IgGI and IgG3, separately and in combination. Boosting each vaccinee’s personal IgG1 titers by 10-fold shifted all the vaccinees into a non-sensitive plateau region of the landscape (**Figure 4B**) where mean FcγRIIa complex formation was significantly higher than baseline (2.85e-10 nM, adjusted p-value = 0.0069). The IgG1-only boost decreased FcγRIIIa complex formation significantly (2.46e-10 nM, adjusted p-value = 0.03). Boosting IgG3 in isolation by 10-fold significantly decreased FcγRIIa by 17.5% (2.28e-10 nM, adjusted p-value< 0.0001) and increased FcγRIIIa by 70.0% (4.71e-10 nM, adjusted p-value< 0.0001) (**Figure 4C**). Interestingly, simultaneously boosting both IgG1 and IgG3 by 10-fold each led to no significant difference in either FcγRIIa or FcγRIIIa complex formation due to the shifting of the vaccinees along the FcγRIIa ≅ FcγRIIIa intersection (**Figure 4D**). Thus, the ratio or balance between IgG1 and IgG3 is more important to the trade-off between FcγRIIa functions and FcγRIIIa functions more so than maximizing both values simultaneously. The statistical significances between boosting interventions are summarized in **Figure 4E**.

### 2.2 Host genetics influence the optimization of effector functions

**Figure 5:**
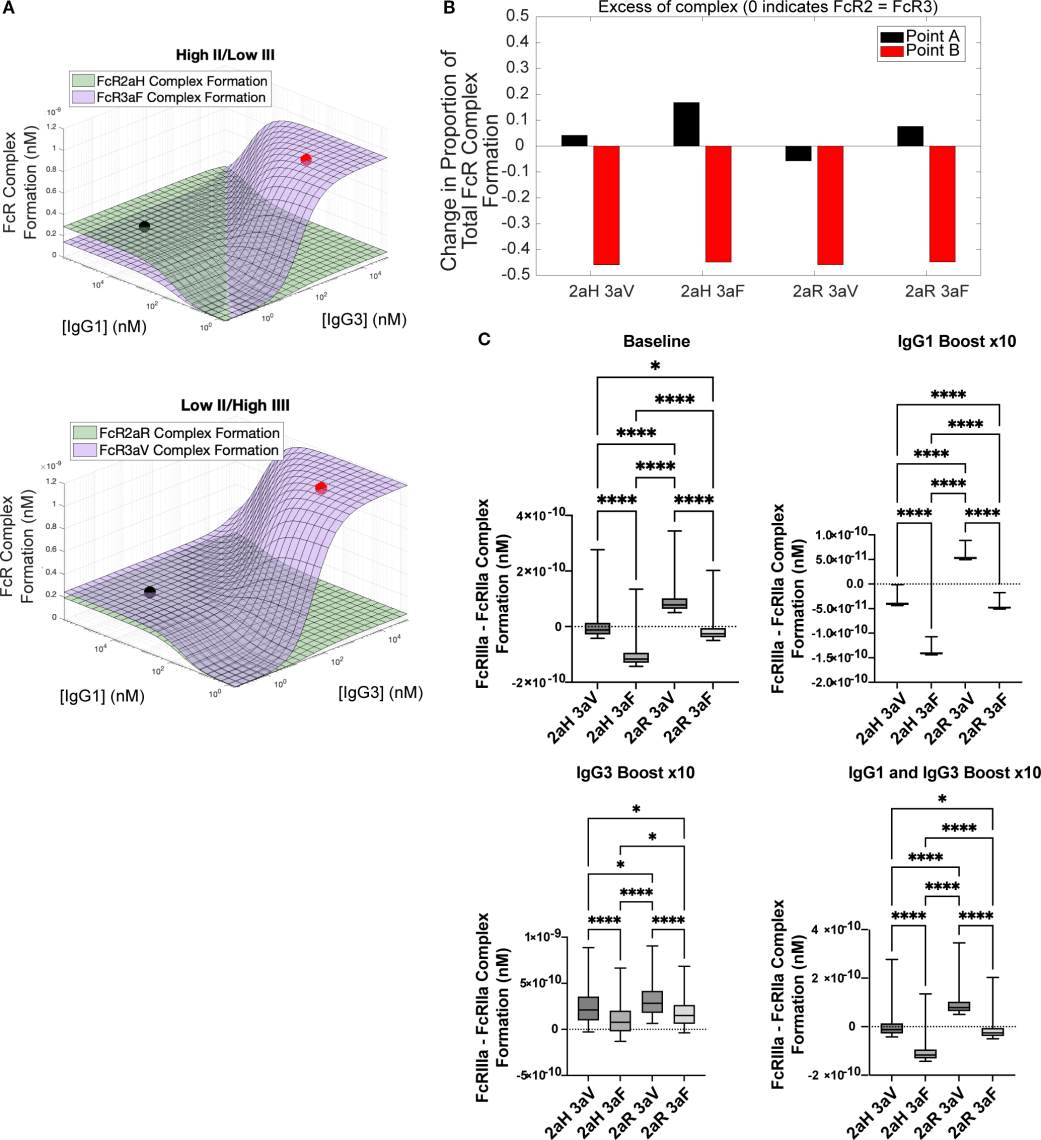
Simulating FcR complex formation across different polymorphisms combinations **(A)** Antibody landscapes for two of the four possible combinations of FcγR genotypes (high-affinity FcγRIIaH131 paired with low affinity FcγRIIIaV158 and low-affinity FcγRIIaR131 paired with high affinity FcγRIIIaV158) show that ADCP has lower potential in the R131/V158 combination. **(B)** Excess complex formation as defined by the formation above 50% correlating with an equal balance of FcγRIIa/IIIa. **(C)** Difference between FcγRIIIa and FcγRIIa complex formation across different boosting conditions for the RV144 vaccinee data. Differences between boosting regimens were evaluated using the Kruskal-Wallis test with Dunn’s multiple comparisons test. (*p < .05, ****p < .0001).

We next applied this quantitative framework to investigate differences predicted to arise from genetic differences in Fc receptor polymorphisms, which have the potential to change IgG binding to FcγRs. We evaluated two known polymorphisms for both FcγRIIa and FcγRIIIa including the higher affinity polymorphisms (FcγRIIa-H131 and FcγRIIIa-V158) as well as the lower affinity polymorphisms (FcγRIIA-R131 and FcγRIIIa-F158) (19). For each polymorphism, we evaluated both homozygous and heterozygous combinations. The landscapes for FcR formation with the two heterozygous combinations (H131/F158 and R131/V158) are shown in **Figure 5A**. With the R131/V158 genotype (low affinity FcγRIIa and high affinity FcγRIIIa), FcγRIIIa is always greater than FcγRIIa regardless of IgG1 and IgG3 levels. There is also a much lower maximum for ADCP (FcγRIIIa) with this combination in comparison to the H131/F158 combination (high affinity FcγRIIa and low affinity FcγRIIIa). Example points A (high IgG1 and low IgG3) and B (high IgG3 and low IgG1) were used to display the balance of FcγRIIa and FcγRIIIa across the four different polymorphism genotype combinations in **Figure 5B**. A horizontal line indicates equivalent FcγRIIa and FcγRIIIa formation; above the line is higher ADCP responses (FcγRIIa), while below the line is higher ADCC (FcγRIIIa) responses. While ADCC responses are relatively consistent across phenotypes, the R131/V158 combination is the only genotype to flip to higher levels of ADCC rather than ADCP at high levels of IgG1.

We next plotted vaccinee data on our polymorphism landscapes in simulated boosting scenarios to determine whether individuals could respond differently to interventions based on FcR polymorphism combinations. Model predictions indicated significant differences for baseline FcγRIIa formation between any combination that included H131 versus R131 (2.76e-10 nM for H131 versus 1.86e-10 nM for R131, adjusted p-value< 0.0001). In contrast, there were no significant differences in FcγRIIa formation with H131 (or R131) when comparing a genetic pairing with F158/V158. We observed a similar pattern for FcγRIIIa formation (2.77e-10 nM for V158 versus 1.71e-10 nM for F158, adjusted p-value< 0.0001). **Figure 5C** summarizes the differences between FcγRIIIa and FcγRIIa formation in all four polymorphism combinations, with the greatest difference observed between R131/V158 (higher ADCC) and H131/F158 (higher ADCP). The same pattern was observed across all of the boosting patterns, including IgG1-only boost, IgG3-only boost, and IgG1 and IgG3 boost. These results indicate that personalized differences in binding affinity based on genetic polymorphism combinations could play an important role in an individual’s capacity for maximizing specific Fc effector functions.

### 2.3 The model illustrates the potential for altered Fc responses in mucosal tissues

**Figure 6:**
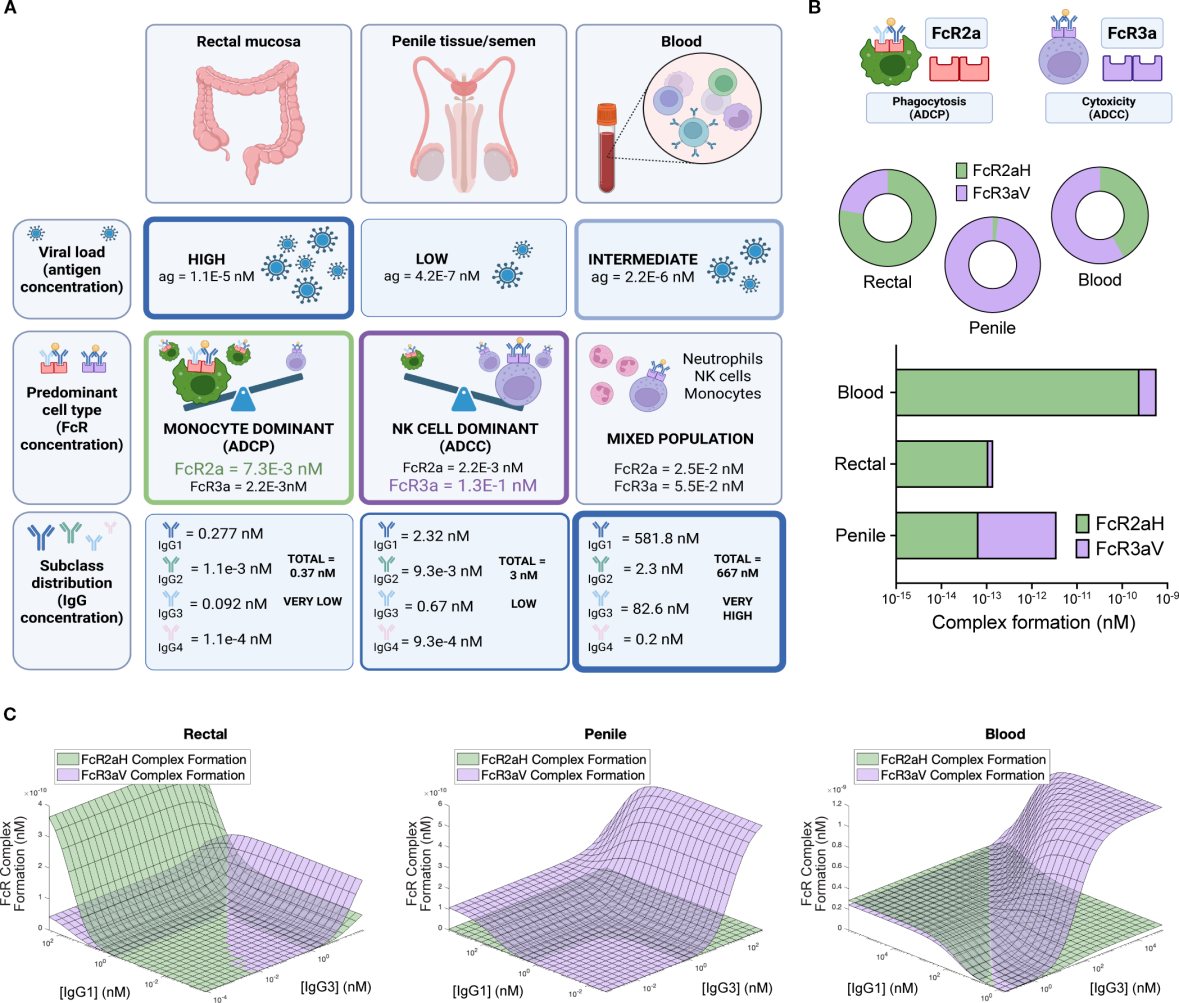
Fc receptor functions across different tissues. **(A)** Visualization of baseline parameters used as input for mucosal (rectal and penile) and blood models. Sources summarized in **Supplementary Tables 1**, **2**. **(B)** Baseline simulation results showing complex formation involving FcγRIIa and FcγRIIIa as log complex formation or percentage of total FcγR complexes. **(C)** 2D landscapes showing IgG1 and IgG3 combinations affect ADCP and ADCC model output as measured by FcγR complex formation.

Fc effector functions play an important role in mucosal tissues (24), however these functions are difficult to evaluate *in vivo*. We used the model to make predictions for how FcR complex formation might change in mucosal tissues as a function of differences in viral load, variability of Fc receptor expression on effector cells, the number of effector cells, and IgG titers present at mucosal sites. We used parameters from the literature as shown in **Supplementary Tables 1**, **2**, and **Figure 6A**. Viral load (antigen concentration) is estimated to be highest in the rectal mucosa, however antibody concentrations are more than 100x lower in mucosal surfaces than in the blood (20, 22, 23, 25, 26). In the blood, there are similar levels of FcγRIIa and FcγRIIIa (42%/58%) (23) but published data suggests that the balance is much more skewed in mucosal tissues (**Figure 6B**). For example, in the rectal tissue there are more monocytes and thus higher expression of FcγRIIa (associated with ADCP; 78% of combined FcγRIIa and FcγRIIIa expression), while in the penile tissue there are more natural killer cells present and thus higher expression of FcγRIIIa (associated with ADCC; 98% of combined FcγRIIa and FcγRIIIa expression).

As expected, due to the larger number of monocytes in the rectal mucosa, the model suggested that ADCP was the more prominent function in this tissue type. The penile tissue and semen had lower levels of antigen, and mostly NK cells which leads to prominence of ADCC. The blood had a mixture of the two functions but has much higher levels of antibodies than the mucosal tissues, leading to overall higher Fc effector responses.

We then used the model to predict FcγRIIa and FcγRIIIa complex formation across variable combinations of IgG1 and IgG3 in penile and rectal tissue compared to blood (**Figure 6C**). Model predictions suggested that in the penile tissue, ADCC was the dominant function and boosts in IgG1 or IgG3 mainly caused increases in ADCC without much change in ADCP. In the rectal tissues, high IgG1 at any level of IgG3 had the potential to result in a steep increase in ADCP, a pattern which was not present in other tissue we evaluated. These results could be informative for the future design of tissue-specific boosting interventions, or for understanding the impact of antibody decay in different tissues. For example, if ADCC is protective against HIV transmission (27–29), the model illustrates how interventions that preferentially boost ADCP (such as increasing IgG1) in the rectal tissue have the potential to detract from more protective ADCC responses. Conversely, increases in IgG1 or IgG3 both have the potential for improving ADCP and ADCC in penile tissue. We additionally explored how polymorphism combinations may affect FcR effector functions in the mucosal tissues. Unlike model predictions in blood, polymorphisms did significantly alter the balance of ADCP and ADCC in mucosal tissues, likely due to considerably lower antibody levels that reduce the sensitivity to FcR-antibody affinity compared to antibody concentrations (**Supplementary Figure 3**).

## 3 Discussion

Overall, this study offers a new quantitative framework to evaluate the complex interplay between antibody-dependent cellular phagocytosis (ADCP) and antibody-dependent cellular cytotoxicity (ADCC) in immune responses. By modeling the interactions of different IgG subclasses with Fc receptors (FcRs), we challenge the conventional view that higher antibody titers necessarily enhance Fc-mediated immune functions. Instead, the model reveals the potential for a more nuanced mechanism, where competition between IgG subclasses for antigen binding and differential affinities for FcRs can lead to an inverse relationship between IgG titers and specific effector functions. This new insight was not apparent in our previous work involving models that focused on single FcR receptors in isolation [18]. This highlights the importance of IgG subclass distribution in shaping immune responses, which may vary across individuals and vaccine regimens. For example, the model demonstrates that high levels of IgG1 can increase ADCP but may suppress ADCC, while elevated IgG3 enhances ADCC but reduces ADCP. This delicate balance suggests that neither ADCP nor ADCC is maximized when both IgG1 and IgG3 are elevated, pointing to an optimal range where these functions can coexist. This insight has significant implications for vaccine development strategies, particularly in the context of personalized medicine. Our findings suggest that even small changes in IgG subclass titers can significantly alter Fc effector functions, especially in individuals whose antibody levels fall within sensitive regions of the functional landscape. Furthermore, simulated boosting of IgG1 and IgG3 does not enhance both ADCP and ADCC as expected, underscoring the complexity of antibody competition and the need for more precise immunomodulation strategies.

This quantitative framework also extends beyond subclass boosting, offering a means to investigate the role of FcR polymorphisms, which are often challenging to assess independently of individual variability in antibody titers. There have been mixed results on the association between FcR polymorphisms and HIV infection with and without vaccines (30). Many of these studies have looked at the effect of homozygous or heterozygous polymorphisms, while also considering the combination across FcR types (high affinity FcγRIIa with low affinity FcγRIIIa). The model created here with two FcRs was also able to be evaluate homozygous versus heterozygous combinations within one FcR type, which produced the expected results that heterozygous individuals tend to fall between the responses in homozygous individuals. FcγRIIa has been proposed as a mediator of latent reservoirs, suggesting that polymorphisms that change its affinity may affect the size of the reservoir (31). Individuals with a low-affinity FcγRIIa/high-affinity FcγRIIIa combination were more likely to exhibit ADCC dominance, potentially making it difficult to optimize ADCP responses in those individuals. Interestingly, we found that these differences were less pronounced in mucosal tissues compared to the blood, likely due to lower antibody concentrations in mucosal tissues. This finding underscores the importance of tissue-specific immune dynamics, which could influence pathogen transmission and disease progression in ways that blood-based models cannot fully capture. Furthermore, the same model can be used to explore how post-translational modifications, such as glycosylation and fucosylation of the antibody Fc region, further modulate these interactions.

The two FcR model created here provides a valuable approach for investigating similar mechanisms in other infectious and autoimmune diseases. For example, Fc-mediated antibody responses are essential in protection against SARS-CoV-2, mediated by both vaccination and natural infection, as they contribute to viral clearance and correlate with outcomes such as disease severity and survival (32–37). Notably, studies on SARS-CoV-2 mRNA vaccines have revealed that boosting may disproportionately increase IgG4 titers, potentially reducing Fc effector functions like ADCP (38). This highlights the importance of considering the variability of subclass concentrations, binding affinities, and Fc functions when designing vaccines and therapeutic strategies for other diseases, where skewed subclass responses or impaired Fc functions might influence disease progression or protection.

There are a number of limitations to the current model that could be modified based on specific future applications. The model considers the described IgG-FcR system in isolation from other receptors, ligands, and antibodies that are certainly present in *in vivo* scenarios. While this presents a unique opportunity to understand the isolated mechanisms that link IgG subclass distribution to FcγRIIa and FcγRIIIa complexes upstream of ADCC and ADCP, the model would require the addition of other FcR and antibody species to capture other Fc effector functions or to better approximate specific *in vivo* conditions. IgA would be essential to include in future models, especially those involving mucosal tissues. We also note several assumptions that were used to simplify the parameters of the model, including the equal affinity of each subclass binding to antigen, and similar dissociation rates for each antibody subclass to FcRs, though these could also be modified in future applications. Lastly, the model uses FcR complex formation as a surrogate for downstream Fc effector functions themselves. This assumption is reasonable given the positive association between FcγRIIa engagement and ADCP and that between FcγRIIIa engagement and ADCC. Nevertheless, it would be important to include downstream signaling and inhibitory receptors in future iterations of the model. It would also be important to validate the model predictions using cellular function assays.

This work supports the future development of multiplex assays that would enable experimental validation of models that include multiple Fcγ receptors in parallel. Existing studies, including those used for the baseline values of boosting studies in **Figure 4**, have been validated using single-receptor systems such as those described in Lemke et al. (2021). However, no published assays currently measure the simultaneous activation or engagement of multiple FcγRs in the same experimental context, independent of downstream signaling. Our model highlights the need for these, to test newly generated ideas regarding competition among IgG subclasses, antigen availability, and FcR binding. The novel predictions emerging from our dual-FcR framework underscore the biological relevance of co-expressed receptors and suggest that experimental systems capable of capturing this complexity are essential for a more complete understanding of Fc effector functions *in vivo*.

Despite its limitations, the model provides an important step towards integrating literature-based parameters to understand mechanistic differences in Fc effector functions across different tissues that are difficult to evaluate and sample experimentally. In the future, this model could be used to optimize antibody responses, tailor vaccinations to individual variability, guide local boosting strategies, and enhance our understanding of immune responses in different tissues to inform the design of novel vaccines and improve immunotherapy outcomes. Despite remaining challenges, this framework represents a promising tool for low-cost hypothesis testing related to immune responses in both the blood and mucosal tissues, offering potential pathways for improved vaccine design and therapeutic interventions.

## 4 Methods

### 4.1 Computational methods

#### 4.1.1 System of ordinary differential equations

We expanded the system of ODE equations established in Lemke et al. (2021) and Lemke et al. (2022) to account for the potential binding of antigen-bound IgG dimers to multiple different classes of FcγRs, thereby considering potential competition between substrates and receptors within the system (17, 18). The env-IgG-FcγRIIa and -FcγRIIIa framework is illustrated in the model schematic with example reaction equation shown in **Figure 1**. The system of equations is written using the laws of mass action kinetics, in combination with conservation equations for total antigen, total FcR, and each IgG subtype. We assumed no degradation or production of species over the short time span of the model.

The model includes stepwise chemical kinetic reactions, beginning with antigen binding and progressing to Fcγ receptor complex formation.

1. Antigen antibody binding: 2 k_on IgG1-ag_ [IgG1][antigen] - k_off IgG1-ag_ [IgG1-antigen]
2. Antibody dimerization: k_on IgG1-ag_ [IgG1][IgG1-antigen] - 2 k_off IgG1-ag_ [IgG1-IgG1-antigen]
3. FcR2aH complex binding: k_on IgG1-FcR2aH_ [FcR2aH][IgG1-IgG1-antigen] - k_off IgG1-FcR2aH_ [FcR2aH-IgG1-IgG1-antigen] FcR3aV complex binding = k_on IgG1-FcR3aV_ [FcR3aV][IgG1-IgG1-antigen] - k_off IgG1-FcR3aV_ [FcR3aV-IgG1-IgG1-antigen]

The system is governed by mass-action kinetics. As an example, the rate of change in the single bound IgG1-antigen complexes is given by:

d[IgG1-antigen]/dt = 2k_on IgG1-ag_ [IgG1][antigen] - k_off IgG1-ag_ [IgG1-antigen]
- k_on IgG1-ag_ [IgG1-antigen][IgG1] + k_off IgG1-ag_ [IgG1-IgG1-antigen]
- k_on IgG2-ag_ [IgG1-antigen][IgG2] + k_off IgG2-ag_ [IgG1-IgG2-antigen]
- k_on IgG3-ag_ [IgG1-antigen][IgG3] + k_off IgG3-ag_ [IgG1-IgG3-antigen]
- k_on IgG4-ag_ [IgG1-antigen][IgG4] + k_off IgG4-ag_ [IgG1-IgG4-antigen]

Each antibody subclass is tracked in a similar manner, resulting in a large but structured system of ODEs (See **Supplementary Material**: Detailed description of the system of ODEs). Total concentrations of each IgG subclass, antigen, and FcR are conserved. For example, the conservation equation for IgG1 is:

Free IgG1 = Total IgG1 - Bound IgG1
[IgG1] = [IgG1_total_] - [IgG1-antigen] - 2 [IgG1-IgG1-antigen]
- [IgG1-IgG2-antigen] - [IgG1-IgG3-antigen]
- [IgG1-IgG4-antigen] - 2 [IgG1-IgG1-antigen-FcR2]
- [IgG1-IgG2-antigen-FcR2] - [IgG1-IgG3-antigen-FcR2]
- [IgG1-IgG4-antigen-FcR2] - 2 [IgG1-IgG1-antigen-FcR3]
- [IgG1-IgG2-antigen-FcR3] - [IgG1-IgG3-antigen-FcR3]
- [IgG1-IgG4-antigen-FcR3]

The initial IgG subclass concentrations were estimated from Raux et al. (2000) (20) based on gp120 antigen-specific IgG1 and IgG3 EU/mL values measured in the serum, seminal secretions, and rectal secretions in HIV-type 1 infected subjects (asymptomatic CDC stage II/III infection). The IgG2 and IgG4 were found to be very low in HIV-infected individuals and were estimated to be 100 EU/mL and 10 EU/mL respectively. The EU/mL values were then converted to estimated concentrations based on total IgG concentration measured in untreated HIV-infected individuals in Pillay et al. (2019) (21) and subsequently using the expected proportions of each subclass in serum, seminal secretions, and rectal secretions to calculate the subclass concentrations across each tissue. The antigen concentrations in each tissue were obtained from Zuckerman et al. (2004) (22), in which HIV RNA levels were measured in untreated HIV-infected individuals in the plasma, rectum, and semen. The RNA levels were converted to nanomolar concentrations assuming 20 envelope proteins on the surface of each virus (39) and then using Avogadro’s number. The FcR concentrations were calculated based on a study by Cheeseman et al. (2016) (23) in which they measured the distribution of CD14+ monocytes and natural killer (NK) cells in whole blood, penile tissue, and colorectal tissue. They then used flow cytometry to characterize the expression of various Fc receptors on the different cell types. We used these values accounting for the distribution of cell types in each tissue and then the expression of FcγRIIa and FcγRIIIa on each cell type to calculate a summed total FcγR concentration for each tissue type. **Supplementary Tables 1**, **2** contain a summary of the affinity and concentration parameter values and sources for the model respectively.

The initial concentration of each complex was set to zero and binding affinities for lgG1, lgG2, lgG3, lgG4, and FcγR dimers were set based on literature values (19). Envelope binding affinities were determined via SPR measurements as described **Supplementary Table 1**. We obtained K_A_s for each IgG subclass binding to FcγRIIIA-V158 from the literature (19) and converted these K_A_s to k_on_s by estimating a universal k_off_ from pooled RV144 serum samples (0.01 s^-1^), as done in Lemke et al. (2021). MATLAB’s ode113 solver function was utilized to predict the concentration of each complex over 10^5^ seconds, with an absolute error tolerance of 1e-50 and a relative tolerance of 1e-10. We assumed sequential IgG antibody binding to antigen prior to engagement of any antigen-IgG-IgG complex with any FcγR dimer. We assumed no cooperativity in IgG binding antigen (such that affinity parameters were independent of the presence of another IgG on the same antigen). For antigen-IgG complexes containing two of the same IgG subclass we used literature values for the reported value of that subclass (19). For complexes containing two different IgG subclasses, we assumed the affinity of the heterogeneous complex to be the average of the two individual IgG subclass affinities

#### 4.1.2 MFI conversion to nM

The de-identified RV144 data (available in GitHub repository) is used in this paper to simulate the effects of boosting personalized IgG1 and/or IgG3 concentration on FcγR complex formation. This data comes from the RV144 phase III clinical trial from the US Military HIV Research Program (MHRP) (40). Samples from week 26 (2 weeks post vaccination) RV144 vaccine recipients (n = 30; n = 75 from two separate shipments) were evaluated using data from McLean et al. (2017) and are the same samples validated using the single FcR model in Lemke et al. (2021) (17, 41). When plotting RV144 vaccinee data for boosting simulations, experimental MFI measurements (as described in Rerks-Ngarm et al. (2009) (7)) were converted to concentration measurements using a conversion factor based on a reference IgG1 concentration of 10,000 ng/mL, as done in previous work by Lemke et al. (2021) (17). For multiplex readings, there is a log-linear relationship between MFI and concentration when measurements are within the machine’s dynamic range (42). Conversion formulas were based on this typical relationship. We assumed that MFI measurements were in the dynamic range, and that the average IgG1 concentration was 10,000 ng/mL. The conversion factor found for IgG1 was then applied to the remaining species within that given assay. The concentrations for species IgGn (where n = 1 through 4) were converted from assay concentrations to plasma concentrations by multiplying by a factor of 200 to account for dilution of plasma samples for the assay.


$$\text{Conversion Factor}=\;\frac{\log_{10}\text{Reference Concentration}}{\text{mean}\left(\log_{10}{\text{MFI}}_{IgG1}\right)}$$

$$\text{[IgGn] in nM}=\;\frac{10^{(\log_{10}{\text{MFI}}_{\text{IgGn}})*\text{Conversion Factor}}}{{\text{Molecular weight}}_{\text{IgGn}}}*200$$

#### 4.1.3 Sensitivity analyses

We first performed a one-dimensional sensitivity analysis across each of the tissues for the two FcγR model. This was done by calculating the model output (FcγRIIa complex formation and FcγRIIIa complex formation) at the baseline parameter value, and then also varying each parameter, one at a time across a physiological relevant range. We use baseline blood concentration values from **Supplementary Table 2** (IgG1 = 581 nM, IgG2 = 2.3 nM, IgG3 = 82.6 nM, IgG4 = 0.23 nM, Antigen = 2.2e-6 nM. FcRII = 2.5e-2 nM, and FcRIII = 5.5e-2) and the affinity parameters from **Supplementary Table 1** and then take each of the 31 baseline parameters multiplied by a factor (0.05x, 0.1x, 0.4x, 2.5x, 10x, and 20x) to simulate a wide range of possible parameters. **Figure 2** displays these results, where each box represents a simulation run with the baseline parameters and one altered parameter, in which we calculate the FcR complex formation output of the model. The heatmap shows the log of the FcR complex formation, such that red indicates higher FcγR complex formation and blue indicates lower FcγR complex formation. Exact values for the sensitivity analyses across blood, penile, and rectal tissues are displayed in **Supplementary Figure 2**.

We also performed a global uncertainty and sensitivity analysis for FcRII and FcRIII complex formation (43) using the baseline blood parameters described above and in **Supplementary Table 1** and the affinity parameters from **Supplementary Table 2**. In the global sensitivity analysis algorithm provided by the Kirschner lab at the University of Michigan, we assigned log-uniform probability density functions (pdfs) to each parameter (initial concentrations and affinities) with a minimum 0.05X of baseline and a max 20X of baseline for all parameters (43). These pdfs were sampled using Latin hypercube sampling (LHS) to create random combinations of parameter values. The model was evaluated under each of the 5,000 sets of random parameter combinations, allowing for a multidimensional exploration of the system. Partial rank correlation coefficient (PRCC) calculated within the algorithm determined the correlation between each input variable’s variance throughout multidimensional analysis and the output variable, giving a sensitivity measure for each parameter and a statistical significance of its effect on complex formation. The results are shown in **Supplementary Figure 1**.

#### 4.1.4 Generation of IgG1 versus IgG3 landscapes

We were also able to perform two-dimensional sensitivity analyses by varying two parameters within the model simultaneously and plotting the results on a landscape surface. 625 simulations were run with differing combinations of initial IgG1 and IgG3 concentrations with tissue-specific baseline values for all other parameters. All combinations of 25 IgG1 and IgG3 concentration values were uniformly spaced on a logarithmic scale between 0.001X-1000X baseline concentrations. Results were plotted as a grid surface for each FcγR, with FcγRIIa (green) and FcγRIIIa (purple) complex formation across the IgG1 and IgG3 combinations plotted on the same graph. Heatmaps showing the complex formation for each FcγR type were also generated (**Figure 3B**). Cross-sections at an intermediate value of IgG1 or IgG3 (100 nM) were visualized as well (**Figure 3C**).

For boosting simulations using RV144 vaccinee data (**Figure 4**), we predicted individual FcγRIIa and FcγRIIIa complex formation (n = 105) based on IgG subclass 1–4 concentrations. This data has been validated using a single FcγR model in Lemke et al. (2021) and is expanded here to visualize the simultaneous formation of both FcγRIIa and FcγRIIIa complexes using the dual receptor model (17). Individuals were plotted as circles at their specific IgG1 and IgG3 initial concentrations, which are converted from MFI to nM as per Section 4.1.2 with their individually predicted complex formation concentration. Simulations of IgG1, IgG3, or simultaneous IgG1 and IgG3 boosting in the dual FcγR model were performed at 10x personal baseline concentrations. Ordinary one-way ANOVA and Tukey’s multiple comparison test were performed to determine significance across the different boosting simulations or FcγR polymorphisms. When evaluating the landscapes for the four different polymorphism combinations, the FcγR polymorphism and IgG class specific affinity parameters were taken from the literature, reported in **Supplementary Table 1**. Example points A and B (using first and third quartile of IgG1 and IgG3 concentrations) were used for demonstration of differences in FcγR complex formation.

#### 4.1.5 Software

ODE modeling, sensitivity analyses, and 3-D plots were completed using MATLAB 2022b (MathWorks, Natick, MA). Visualization of the remaining plots, and statistics were completed using GraphPad Prism version 10. Custom MATLAB code is available (github.com/suzshoff/2FcRODEModel/) to run the simulations necessary to generate the data and figures (steady state complex formation concentrations) used in this analysis.

#### 4.1.6 Statistical analysis

Statistical significance for the IgG boosting vaccinee simulations and polymorphism simulations were determined using a Kruskal-Wallis test with Dunn’s multiple comparisons test (α = 0.05). Statistical significance denoted with adjusted p-value less than 0.05 shown with one asterisk, less than 0.01 with two asterisks, continuing down to less than 0.0001 with four asterisks.

## Data Availability

The original contributions presented in the study are included in the article/**Supplementary Material**. Further inquiries can be directed to the corresponding author.
