# Supplementary material for: Understanding mechanistic relationships between IgG titers and Fc effector functions: a computational framework to assess polyfunctionality
Source: Front Immunol. 2025 Sep 16;16:1578500. doi: 10.3389/fimmu.2025.1578500 (PMC12479434; doi:10.3389/fimmu.2025.1578500)
Supplement: Supplementary file 1 [file DataSheet1.docx]

Supplementary Material

Understanding mechanistic relationships between IgG titers and Fc effector functions: a computational framework to assess polyfunctionality

Suzanne K. Shoffner-Beck^1±^, Robert M. Theisen^1±^, Kade E. Wong^1^, Supachai Rerks-Ngarm^2^, Punnee Pitisuttithum^3^, Sorachai Nitayaphan^4^, Stephen J Kent^5,6^, Amy W. Chung^5^, Kelly B. Arnold^1*^

± Co-first authors

**^1^ Department of Biomedical Engineering, University of Michigan, Ann Arbor, MI, USA**

**^2^ Department of Disease Control, Ministry of Public Health, Bangkok, Thailand.**

**^3^ Vaccine Trial Centre, Faculty of Tropical Medicine, Mahidol University, Bangkok, Thailand.**

**^4^ Armed Forces Research Institute of Medical Sciences, Bangkok, Thailand.**

**^5^ Department of Microbiology and Immunology, The Peter Doherty Institute for Infection and Immunity, The University of Melbourne, Melbourne, Victoria, Australia**

**^6^ Melbourne Sexual Health Centre and Department of Infectious Diseases, Alfred Health, Central Clinical School, Monash University, Melbourne, VIC 3004, Australia**

*** Correspondence:**Kelly Arnold
kbarnold@umich.edu


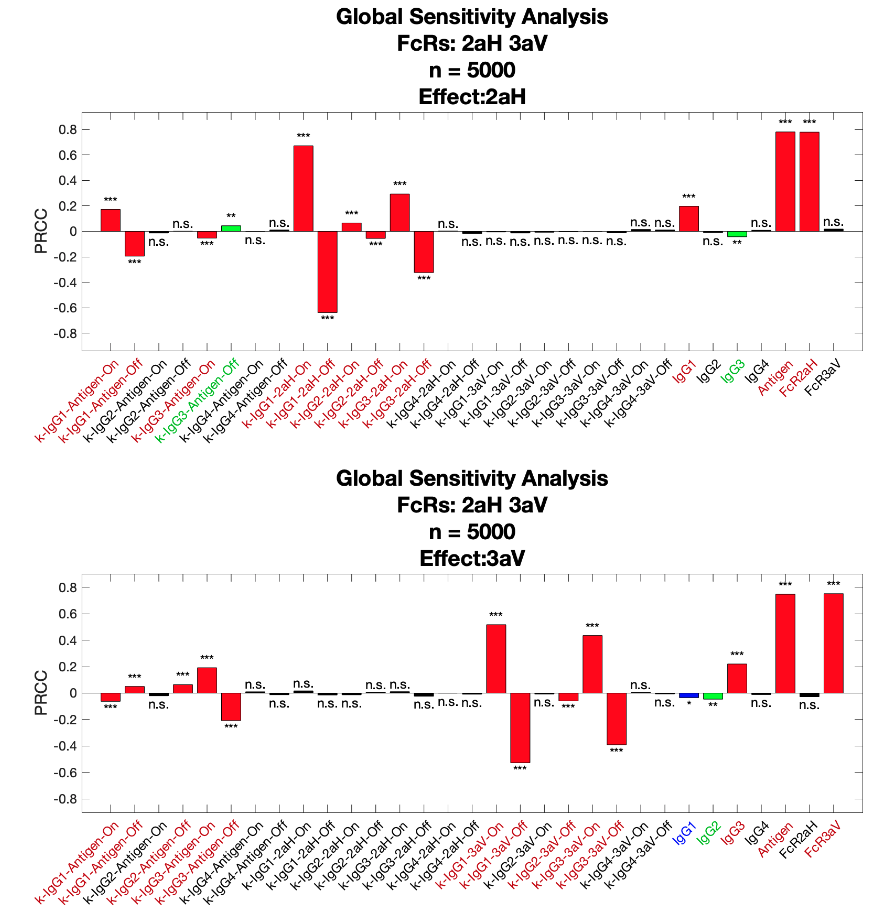


**Supplementary Figure 1:** Global sensitivity analysis using in vivo blood parameters as baseline values

**Supplementary Figure 2**: 1D Sensitivity analysis across blood and mucosal tissues.


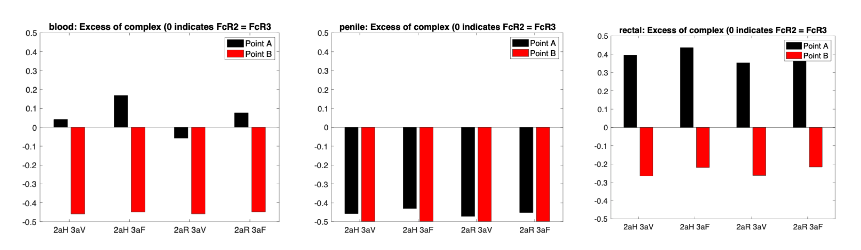


**Supplementary Figure 3**: Genetic polymorphisms across tissues

**Supplementary Table 1**: Affinity parameters for two FcR ODE model

| **FcRII Parameters** | **Value** | **FcRIII Parameters** | **Value** | **Units** | **Source** |
| --- | --- | --- | --- | --- | --- |
| k_on IgG1-FcRIIaH_ | 52e-6 | k_on IgG1-FcRIIIaV_ | 20e-6 | nM^-1^s^-1^ | k_on_ and k_off_ calculated from K_a_ measured in  Bruhns et al. (2009) (18) |
| k_on IgG2-FcRIIaH_ | 4.5e-6 | k_on IgG2-FcRIIIaV_ | 0.7e-6 | nM^-1^s^-1^ |  |
| k_on IgG3-FcRIIaH_ | 8.9e-6 | k_on IgG3-FcRIIIaV_ | 98e-6 | nM^-1^s^-1^ |  |
| k_on IgG4-FcRIIaH_ | 1.7e-6 | k_on IgG4-FcRIIIaV_ | 2.5e-6 | nM^-1^s^-1^ |  |
| k_on IgG1-FcRIIaR_ | 35e-6 | k_on IgG1-FcRIIIaF_ | 11.7e-6 | nM^-1^s^-1^ |  |
| k_on IgG2-FcRIIaR_ | 1e-6 | k_on IgG2-FcRIIIaF_ | 0.3e-6 | nM^-1^s^-1^ |  |
| k_on IgG3-FcRIIaR_ | 9.1e-6 | k_on IgG3-FcRIIIaF_ | 77e-6 | nM^-1^s^-1^ |  |
| k_on IgG4-FcRIIaR_ | 2.1e-6 | k_on IgG4-FcRIIIaF_ | 2e-6 | nM^-1^s^-1^ |  |
| **General Parameters** | | **Value** | | **Units** | **Source** |
| k_off IgG1-4-FcRII/III_ | | 0.01 | | s^-1^ | Average value measured in pooled RV144 plasma for FcRIIIaV (17) |
| k_on IgG1-4-ag_ | | 10e-6 | | nM^-1^s^-1^ | SPR measurements from pooled IgG from HIV individuals (17) |
| k_off IgG1-4-ag_ | | 2e-4 | | s^-1^ |  |

**Supplementary Table 2**: Concentration parameters for two FcR ODE model

| **Parameter** | **Blood value** | **Penile value** | **Rectal value** | **Units** | **Sources** |
| --- | --- | --- | --- | --- | --- |
| IgG1 total | 581 | 2.32 | 0.277 | nM | Subclass proportions/concentrations estimated from Raux et al. (2000) (19) and  Pillay et al. (2019) (20) |
| IgG2 total | 2.3 | 0.0093 | 0.0011 |  |  |
| IgG3 total | 82.6 | 0.67 | 0.092 |  |  |
| IgG4 total | 0.23 | 0.00093 | 0.00011 |  |  |
| Antigen total | 2.2e-6 | 4.2e-7 | 1.1e-5 |  | Zuckerman et al. (2004) (21) |
| FcRII total | 2.5e-2 | 2.2e-3 | 7.3e-3 |  | Cheeseman et al. (2016) (22) |
| FcRIII total | 5.5e-2 | 1.1e-1 | 2.2e-3 |  |  |

**Detailed description of the system of ODEs and full list of equations:**

This system of ordinary differential equations (ODEs) models the interactions between four immunoglobulin G (IgG) subclasses (IgG1, IgG2, IgG3, IgG4) and an antigen (Ag), as well as their binding to Fc receptors FcγRIIa and FcγRIIIa. The variables and parameters describe binding dynamics, formation of immune complexes, and receptor interactions.

**State Variables and Parameters:**

- y(i) represents concentrations of free and bound antibody-antigen complexes
  - igg[1-4]a represents a single IgG bound to antigen
  - igg[1-4]a[1-4] represents two IgGs (IgG1-4 in various combinations) bound to antigen
  - igg[1-4]a[1-4]r2a represents an immune complex of an IgG combination dimer bound to antigen bound to FcγRIIa
  - igg[1-4]a[1-4]r3a represents an immune complex of an IgG combination dimer bound to antigen bound to FcγRIIIa
  - The differential equations for each of these complexes is denoted as d[1-4]a for igg[1-4]a, d[1-4]a[1-4] for igg[1-4]a[1-4], etc.
- p(i) are rate constants governing binding and dissociation events.
  - Forward and reverse (k_[1-4]af and k_[1-4]ar) rate constants describe binding/dissociation for each IgG[1-4] to antigen.
  - Additional forward and reverse (k_[1-4]r[2-3]f, k_[1-4]r[2-3]r) rate constants represent FcγRIIa [2] and FcγRIIIa [3] binding to IgG[1-4]-antigen complexes.
  - When an FcR complex is formed with two different subclasses of IgG, the average of the binding rates for each subclass to FcR is assumed
- The total available IgG subclasses (igg1tot, igg2tot, igg3tot, igg4tot), antigen (agtot), and receptors (fcr2atot, fcr3atot) are conserved.
- In mixed IgG/antigen complexes (i.e. ant-IgG1-IgG2), binding to FcR is assumed to be the mean value of the relevant IgGs.

Below find the full set of equations and the corresponding parameters used, as summarized in Supplemental Tables 1 & 2 above:

k1af = p(1);

k1ar = p(2);

k2af = p(3);

k2ar = p(4);

k3af = p(5);

k3ar = p(6);

k4af = p(7);

k4ar = p(8);

k1r2f = p(9);

k1r2r = p(10);

k2r2f = p(11);

k2r2r = p(12);

k3r2f = p(13);

k3r2r = p(14);

k4r2f = p(15);

k4r2r = p(16);

k1r3f = p(17);

k1r3r = p(18);

k2r3f = p(19);

k2r3r = p(20);

k3r3f = p(21);

k3r3r = p(22);

k4r3f = p(23);

k4r3r = p(24);

igg1tot = p(25);

igg2tot = p(26);

igg3tot = p(27);

igg4tot = p(28);

agtot = p(29);

fcr2atot = p(30);

fcr3atot = p(31);

igg1a = y(1);

igg2a = y(2);

igg3a = y(3);

igg4a = y(4);

igg1a1 = y(5);

igg1a2 = y(6);

igg1a3 = y(7);

igg1a4 = y(8);

igg2a2 = y(9);

igg2a3 = y(10);

igg2a4 = y(11);

igg3a3 = y(12);

igg3a4 = y(13);

igg4a4 = y(14);

igg1a1r2a = y(15);

igg1a2r2a = y(16);

igg1a3r2a = y(17);

igg1a4r2a = y(18);

igg2a2r2a = y(19);

igg2a3r2a = y(20);

igg2a4r2a = y(21);

igg3a3r2a = y(22);

igg3a4r2a = y(23);

igg4a4r2a = y(24);

igg1a1r3a = y(25);

igg1a2r3a = y(26);

igg1a3r3a = y(27);

igg1a4r3a = y(28);

igg2a2r3a = y(29);

igg2a3r3a = y(30);

igg2a4r3a = y(31);

igg3a3r3a = y(32);

igg3a4r3a = y(33);

igg4a4r3a = y(34);

**Conservation equations**

ag = agtot - igg1a - igg2a - igg3a - igg4a...

- igg1a1 - igg1a2- igg1a3 - igg1a4...

- igg2a2 - igg2a3 - igg2a4...

- igg3a3 - igg3a4...

- igg4a4...

- igg1a1r2a - igg1a2r2a - igg1a3r2a - igg1a4r2a...

- igg2a2r2a - igg2a3r2a - igg2a4r2a...

- igg3a3r2a - igg3a4r2a...

- igg4a4r2a...

- igg1a1r3a - igg1a2r3a - igg1a3r3a - igg1a4r3a...

- igg2a2r3a - igg2a3r3a - igg2a4r3a...

- igg3a3r3a - igg3a4r3a...

- igg4a4r3a;

igg1 = igg1tot - igg1a - 2*igg1a1 - igg1a2 - igg1a3 - igg1a4...

- 2*igg1a1r2a - igg1a2r2a - igg1a3r2a - igg1a4r2a...

- 2*igg1a1r3a - igg1a2r3a - igg1a3r3a - igg1a4r3a;

igg2 = igg2tot - igg2a - igg1a2 - 2*igg2a2 - igg2a3 - igg2a4...

- igg1a2r2a - 2*igg2a2r2a - igg2a3r2a - igg2a4r2a...

- igg1a2r3a - 2*igg2a2r3a - igg2a3r3a - igg2a4r3a;

igg3 = igg3tot - igg3a - igg1a3 - igg2a3 - 2*igg3a3 - igg3a4...

- igg1a3r2a - igg2a3r2a - 2*igg3a3r2a - igg3a4r2a...

- igg1a3r3a - igg2a3r3a - 2*igg3a3r3a - igg3a4r3a;

igg4 = igg4tot - igg4a - igg1a4 - igg2a4 - igg3a4 - 2*igg4a4...

- igg1a4r2a - igg2a4r2a - igg3a4r2a - 2*igg4a4r2a...

- igg1a4r3a - igg2a4r3a - igg3a4r3a - 2*igg4a4r3a;

fcr2a = fcr2atot...

- igg1a1r2a - igg1a2r2a - igg1a3r2a - igg1a4r2a...

- igg2a2r2a - igg2a3r2a - igg2a4r2a...

- igg3a3r2a - igg3a4r2a...

- igg4a4r2a;

fcr3a =fcr3atot...

- igg1a1r3a - igg1a2r3a - igg1a3r3a - igg1a4r3a...

- igg2a2r3a - igg2a3r3a - igg2a4r3a...

- igg3a3r3a - igg3a4r3a...

- igg4a4r3a;

**Differential Equations:**

d1a = 2*k1af*igg1*ag - k1ar*igg1a...

- k1af*igg1a*igg1 + 2*k1ar*igg1a1...

- k2af*igg1a*igg2 + k2ar*igg1a2...

- k3af*igg1a*igg3 + k3ar*igg1a3...

- k4af*igg1a*igg4 + k4ar*igg1a4;

d2a = 2*k2af*igg2*ag - k2ar*igg2a...

- k1af*igg2a*igg1 + k1ar*igg1a2...

- k2af*igg2a*igg2 + 2*k2ar*igg2a2...

- k3af*igg2a*igg3 + k3ar*igg2a3...

- k4af*igg2a*igg4 + k4ar*igg2a4;

d3a = 2*k3af*igg3*ag - k3ar*igg3a...

- k1af*igg3a*igg1 + k1ar*igg1a3...

- k2af*igg3a*igg2 + k2ar*igg2a3...

- k3af*igg3a*igg3 + 2*k3ar*igg3a3...

- k4af*igg3a*igg4 + k4ar*igg3a4;

d4a = 2*k4af*igg4*ag - k4ar*igg4a...

- k1af*igg4a*igg1 + k1ar*igg1a4...

- k2af*igg4a*igg2 + k2ar*igg2a4...

- k3af*igg4a*igg3 + k3ar*igg3a4...

- k4af*igg4a*igg4 + 2*k4ar*igg4a4;

d1a1 = k1af*igg1a*igg1 - 2*k1ar*igg1a1...

- k1r2f*igg1a1*fcr2a + k1r2r*igg1a1r2a...

- k1r3f*igg1a1*fcr3a + k1r3r*igg1a1r3a;

d1a2 = k2af*igg1a*igg2 - k2ar*igg1a2...

+ k1af*igg2a*igg1 - k1ar*igg1a2...

- mean([k1r2f k2r2f])*igg1a2*fcr2a + mean([k1r2r k2r2r])*igg1a2r2a...

- mean([k1r3f k2r3f])*igg1a2*fcr3a + mean([k1r3r k2r3r])*igg1a2r3a;

d1a3 = k3af*igg1a*igg3 - k3ar*igg1a3...

+ k1af*igg3a*igg1 - k1ar*igg1a3...

- mean([k1r2f k3r2f])*igg1a3*fcr2a + mean([k1r2r k3r2r])*igg1a3r2a...

- mean([k1r3f k3r3f])*igg1a3*fcr3a + mean([k1r3r k3r3r])*igg1a3r3a;

d1a4 = k4af*igg1a*igg4 - k4ar*igg1a4...

+ k1af*igg4a*igg1 - k1ar*igg1a4...

- mean([k1r2f k4r2f])*igg1a4*fcr2a + mean([k1r2r k4r2r])*igg1a4r2a...

- mean([k1r3f k4r3f])*igg1a4*fcr3a + mean([k1r3r k4r3r])*igg1a4r3a;

d2a2 = k2af*igg2a*igg2 - 2*k2ar*igg2a2...

- k2r2f*igg2a2*fcr2a + k2r2r*igg2a2r2a...

- k2r3f*igg2a2*fcr3a + k2r3r*igg2a2r3a;

d2a3 = k3af*igg2a*igg3 - k3ar*igg2a3...

+ k2af*igg3a*igg2 - k2ar*igg2a3...

- mean([k2r2f k3r2f])*igg2a3*fcr2a + mean([k2r2r k3r2r])*igg2a3r2a...

- mean([k2r3f k3r3f])*igg2a3*fcr3a + mean([k2r3r k3r3r])*igg2a3r3a;

d2a4 = k4af*igg2a*igg4 - k4ar*igg2a4...

+ k2af*igg4a*igg2 - k2ar*igg2a4...

- mean([k2r2f k4r2f])*igg2a4*fcr2a + mean([k2r2r k4r2r])*igg2a4r2a...

- mean([k2r3f k4r3f])*igg2a4*fcr3a + mean([k2r3r k4r3r])*igg2a4r3a;

d3a3 = k3af*igg3a*igg3 - 2*k3ar*igg3a3...

- k3r2f*igg3a3*fcr2a + k3r2r*igg3a3r2a...

- k3r3f*igg3a3*fcr3a + k3r3r*igg3a3r3a;

d3a4 = k4af*igg3a*igg4 - k4ar*igg3a4...

+ k3af*igg4a*igg3 - k3ar*igg3a4...

- mean([k3r2f k4r2f])*igg3a4*fcr2a + mean([k3r2r k4r2r])*igg3a4r2a...

- mean([k3r3f k4r3f])*igg3a4*fcr3a + mean([k3r3r k4r3r])*igg3a4r3a;

d4a4 = k4af*igg4a*igg4 - 2*k4ar*igg4a4...

- k4r2f*igg4a4*fcr2a + k4r2r*igg4a4r2a...

- k4r3f*igg4a4*fcr3a + k4r3r*igg4a4r3a;

d1a1r2a = k1r2f*igg1a1*fcr2a - k1r2r*igg1a1r2a;

d1a2r2a = mean([k1r2f k2r2f])*igg1a2*fcr2a - mean([k1r2r k2r2r])*igg1a2r2a;

d1a3r2a = mean([k1r2f k3r2f])*igg1a3*fcr2a - mean([k1r2r k3r2r])*igg1a3r2a;

d1a4r2a = mean([k1r2f k4r2f])*igg1a4*fcr2a - mean([k1r2r k4r2r])*igg1a4r2a;

d2a2r2a = k2r2f*igg2a2*fcr2a - k2r2r*igg2a2r2a;

d2a3r2a = mean([k2r2f k3r2f])*igg2a3*fcr2a - mean([k2r2r k3r2r])*igg2a3r2a;

d2a4r2a = mean([k2r2f k4r2f])*igg2a4*fcr2a - mean([k2r2r k4r2r])*igg2a4r2a;

d3a3r2a = k3r2f*igg3a3*fcr2a - k3r2r*igg3a3r2a;

d3a4r2a = mean([k3r2f k4r2f])*igg3a4*fcr2a - mean([k3r2r k4r2r])*igg3a4r2a;

d4a4r2a = k4r2f*igg4a4*fcr2a - k4r2r*igg4a4r2a;

d1a1r3a = k1r3f*igg1a1*fcr3a - k1r3r*igg1a1r3a;

d1a2r3a = mean([k1r3f k2r3f])*igg1a2*fcr3a - mean([k1r3r k2r3r])*igg1a2r3a;

d1a3r3a = mean([k1r3f k3r3f])*igg1a3*fcr3a - mean([k1r3r k3r3r])*igg1a3r3a;

d1a4r3a = mean([k1r3f k4r3f])*igg1a4*fcr3a - mean([k1r3r k4r3r])*igg1a4r3a;

d2a2r3a = k2r3f*igg2a2*fcr3a - k2r3r*igg2a2r3a;

d2a3r3a = mean([k2r3f k3r3f])*igg2a3*fcr3a - mean([k2r3r k3r3r])*igg2a3r3a;

d2a4r3a = mean([k2r3f k4r3f])*igg2a4*fcr3a - mean([k2r3r k4r3r])*igg2a4r3a;

d3a3r3a = k3r3f*igg3a3*fcr3a - k3r3r*igg3a3r3a;

d3a4r3a = mean([k3r3f k4r3f])*igg3a4*fcr3a - mean([k3r3r k4r3r])*igg3a4r3a;

d4a4r3a = k4r3f*igg4a4*fcr3a - k4r3r*igg4a4r3a;

|  |
| --- |
| **Supplementary Figure 4:** Baseline model time course for formation of FcRIIaH and FcRIIIaV complexes using baseline blood parameters from **Supplementary Table 2** |

| 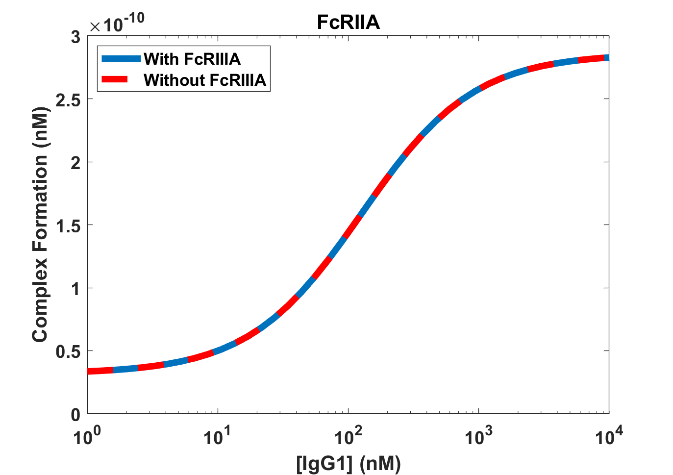 | 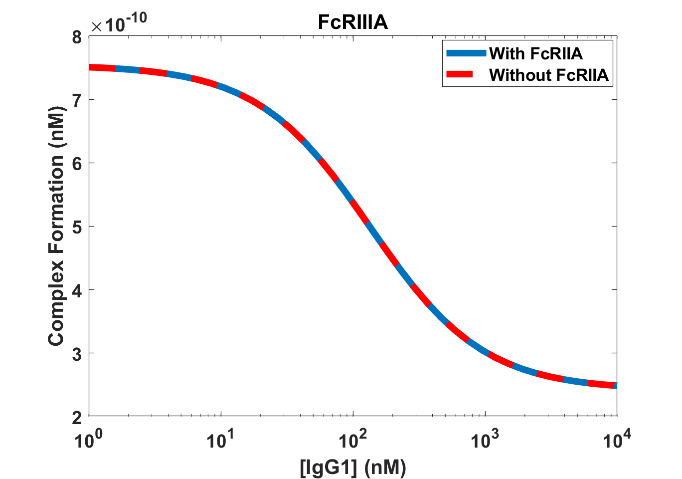 |
| --- | --- |
| **Supplementary Figure 5:** FcRIIa (left) and FcRIIIa (right) complex formation was computed as IgG1 was increased in the two-receptor model with (blue) and without the presence of the other receptor (red; similar to one-receptor model in Lemke et al., 2021)). These simulations were performed using baseline parameters blood. (Supplementary Tables 1 and 2) and show no difference between the one- or two- receptor model for either FcRIIA or FcRIIIA. | |

| 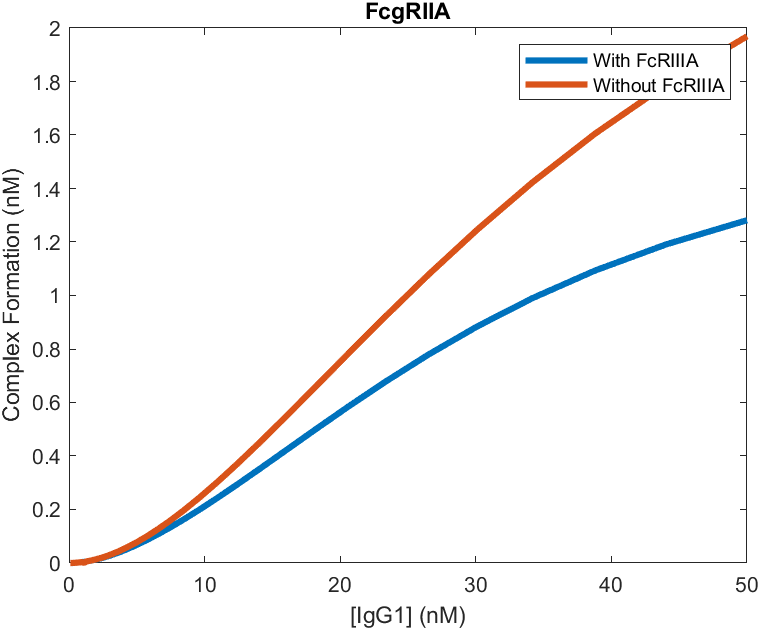 | 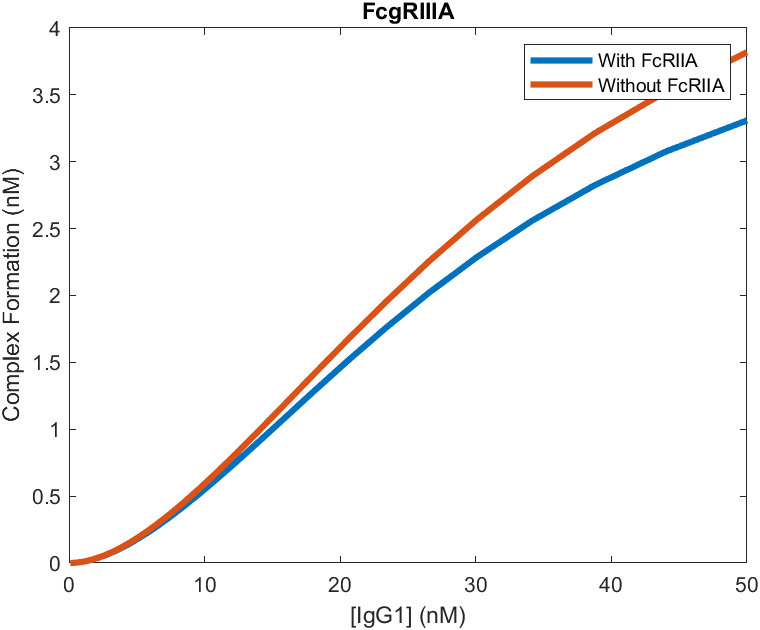 |
| --- | --- |
| **Supplementary Figure 6:** Complex formation for each receptor was computed in the model with and without the presence of the other receptor using blood parameters, but with an increased concentration of antigen (10nM) and FcRs (300 nM). In this parameter space, competition between FcRs has the potential to influence complex formation. | |
